# Interspecific attraction between ground-nesting songbirds and ants: the role of nest-site selection

**DOI:** 10.1186/s12983-021-00429-6

**Published:** 2021-09-10

**Authors:** Marta Maziarz, Richard K. Broughton, Luca Pietro Casacci, Grzegorz Hebda, István Maák, Gema Trigos-Peral, Magdalena Witek

**Affiliations:** 1 Museum and Institute of Zoology, Polish Academy of Sciences, Wilcza 64, 00-679 Warsaw, Poland; 2 UK Centre for Ecology & Hydrology, Maclean Building, Benson Lane, Crowmarsh Gifford, Wallingford, OX10 8BB UK; 3 Department of Life Sciences and Systems Biology, University of Turin, Via Accademia Albertina 13, 10123 Turin, Italy; 4 Institute of Biology, University of Opole, Oleska 22, 45-052 Opole, Poland; 5 Department of Ecology, University of Szeged, Közép fasor 52, Szeged, 6726 Hungary

**Keywords:** Ecological networks, Interspecific interactions, Microclimate, Nest-site selection, Primeval Białowieża forest, Rainfall, Reproduction, Temperature, Weather

## Abstract

**Background:**

Interspecific interactions within ecological networks can influence animal fitness and behaviour, including nest-site selection of birds and ants. Previous studies revealed that nesting birds and ants may benefit from cohabitation, with interspecific attraction through their nest-site choice, but mutual interactions have not yet been tested. We explored a previously undescribed ecological link between ground-nesting birds and ants raising their own broods (larvae and pupae) within the birds’ nests in a temperate primeval forest of lowland Europe. We tested whether the occurrence of ant broods within bird nests resulted from a mutual or one-sided interspecific attraction that operated through nest-site choice and was modified by weather conditions.

**Results:**

We found a non-random occupation of bird nests by ants raising their own broods within them, which indicated interspecific attraction driven solely by the ants. The birds’ preference to nest near tussocks of vegetation showed little overlap with the most frequent placement of ant colonies among fallen deciduous tree-leaves, dead wood and moss. Additionally, birds did not appear to select forest localities with high densities of ant colonies. The occurrence of ant broods within bird nests was also unrelated to bird nest placement near to specific habitat features. The attractiveness of bird nests to ants appeared to increase with the thermal activity of the birds warming their nests, and also during cool and wet weather when the occurrence of ant broods within bird nests was most frequent. Ants often remained in the nests after the birds had vacated them, with only a slight reduction in the probability of ant brood occurrence over time.

**Conclusions:**

The natural patterns of bird nest colonisation by ants support the hypothesis of ants’ attraction to warm nests of birds to raise their broods under advantageous thermal conditions. Similar relationships may occur between other warm-blooded, nest-building vertebrates and nest-dwelling invertebrates, which depend on ambient temperatures. The findings advance our understanding of these poorly recognised interspecific interactions, and can inform future studies of ecological networks.

**Supplementary Information:**

The online version contains supplementary material available at 10.1186/s12983-021-00429-6.

## Background

Species have co-evolved to exist within intricate ecological networks of interspecific interactions, which have shaped their behaviour and life histories [1]. This concept of ecological networks originates from Charles Darwin’s description of the ‘entangled bank’, with its community of interrelated plants, birds, insects and soil biota [2]. Ecological networks have since become a major research topic in ecology, and have been broadly classified into three categories comprising food webs, host-parasite interactions and mutualistic networks [3].

Currently, most ecological networks throughout the World are facing significant and increasing anthropogenic pressures, operating through the destruction or large-scale modification of natural habitats, rapid species decline or extinction, the spread of invasive species and over-arching climate change [3–5]. Due to the complexity of these networks, assessing the effects of anthropogenic disturbances is challenging and requires a good knowledge of the extent and strength of ecological links between species [3, 6]. In this context, research from primeval habitats is particularly valuable for recognising the nature of ecological links unaffected by direct impacts of human activity; such environments may reveal important relationships that might have gone undetected in more disturbed habitats.

The interspecific interactions within ecological networks can have a significant influence on animal fitness and behaviour, such as habitat choice and selection of breeding sites. For instance, among birds, to promote their breeding success, individuals can choose nesting locations that are as inaccessible to predators as possible, they may camouflage their nests or broods, or nest close to more aggressive species that act as a shield against potential enemies, and create an enemy free space [7]. An example of the latter positive relationship may be seen between birds and ants.

Previous studies showed that birds increased their nest safety by nesting nearby colonies of aggressive ant species; these observations included woodpeckers (Picidae) breeding inside the nests of *Crematogaster* ants, and various tropical bird species nesting close to *Oecophylla* ant colonies, or placing their nests in acacia bushes containing *Pseudomyrmex* ant colonies (reviewed in [8]). In Sweden, different tit species (Paridae) preferentially used nest-boxes attached to trees that hosted foraging *Formica aquilonia* ants, but only where the risk of nest predation by birds and mammals was high [9]. Another advantage for birds nesting nearby ant colonies could be reduced nest infestation by ectoparasites or other invertebrates that are vectors of pathogens [10–13].

Close association between nesting birds and ants in ecological networks may also provide opportunities for ants to benefit from access to bird nests. Bird nests are often composed of insulative materials and are warmed from within by their owners when brooding eggs or chicks and maintaining their own body temperature [14, 15]. As such, these warm nests may be a resource of a warm microclimate valuable for the survival, growth and development of arthropods, including ant larvae or pupae [16–20]. Moreover, as bird nests can be inhabited by numerous other invertebrates, and contain animal debris, these places can supply a rich resource of protein food that may be important for ant broods [18, 21].

Thus, the relationship between nesting birds and ants may be beneficial for one or both parties through improved reproductive performance, and so it may lead to one-way or mutual attraction between these two groups of common terrestrial animals. A large niche overlap between ants and nesting birds could favour their cohabitation, which may potentially be widespread, but the evidence to confirm or refute this is currently lacking. Although ants are well-known for forming numerous mutualistic relationships with plants and other invertebrates [22, 23], positive interactions with nesting birds or other vertebrates have gained much less attention (see above).

To fill this knowledge gap, we explored the poorly known phenomenon of the presence of ant larvae or pupae, and their associated workers, within the structure of bird nests [12, 24, 25]. We conducted the study within a remnant of lowland temperate primeval forest in Europe, where ecological networks have been least disturbed by direct human activity [26, 27]. We hypothesised that the cohabitation between nesting birds and ants reflects a mutual attraction, and is an overlooked ecological link between these two groups. We assumed that the presence of ant larvae or pupae within bird nests would depend both on the birds’ choice to place their nests close to ant colonies, and also the decisions of ant workers to relocate their broods into these nearby bird nests. As many ants have limited mobility when relocating their broods (larvae or pupae) to new locations [28], their colonisation of bird nests would be feasible only if the nests are situated within a reachable distance.

We expected that if an attraction exists between birds and ants, then the presence of ant larvae or pupae within bird nests would be a non-random phenomenon. If birds were attracted to nesting near ant colonies, or their nest site preferences overlapped with those of ants, the birds should select nest-sites close to specific habitat features where ants also place their broods, and/or the birds would select forest localities with relatively high densities of ant colonies. Such nest site selection by birds would be expected to result in more frequent colonisation of bird nests by ants. Alternatively, a non-random occurrence of ant broods within bird nests could reflect a one-way attraction of ants to bird nests.

We hypothesised that ants would colonise bird nests to raise their own broods under more advantageous, warmer conditions than within their own nests elsewhere, as demonstrated previously [20]. As such, we assumed that ant broods would occur within bird nests most frequently in the late nestling period, when large chicks warm the nests most intensively, and when the temperature disparities between the birds’ and the ants’ own nests are greatest [20]. The colonisation of bird nests by ants would be also more frequent during cool and wet weather, when the microclimate of the ants’ own nests, which are reliant on ambient temperatures and solar radiation alone, would be less suitable for raising ant broods than in the warm nests of birds [16, 17, 29, 30]. Conversely, we presumed that ant broods would become less common in the bird nests that were inspected long after they were vacated by the birds, when the thermal conditions had deteriorated [20].

This study is the first to explore the mutual attraction between ground-nesting songbirds and ants within an ecological network in an undisturbed forest ecosystem. The findings advance our understanding of the natural patterns of ants colonising bird nests to rear their own broods within them. The study also provides valuable information of the poorly known interactions between nesting birds and nest-dwelling invertebrates, which can inform future studies of ecological networks.

## Methods

### Study area

To assess the unbiased variation in the prevalence of ant broods in bird nests, we conducted the study in one of the last fragments of temperate primeval forest in lowland Europe. Such old-growth stands have been preserved in the extensive Białowieża Forest (c. 1500 km^2^) which straddles the Polish-Belarusian border. The regional climate is subcontinental with annual mean temperatures during May–July of 13–18 °C, and mean annual precipitation ranging between 426 and 940 mm [31, 32]. The altitude ranges from 134–140 m to 200 m a.s.l. [27].

The best-preserved stands are strictly protected within the Białowieża National Park (hereafter BNP; coordinates of Białowieża village: 52°42′ N, 23°52′ E), where species richness is high and the communities’ structures, interspecific interactions and natural processes have been little affected by direct human activity. Conducting the study in this forest offered a unique opportunity to observe the behaviour of birds and ants under conditions that likely prevailed across lowland Europe before widespread deforestation and forest exploitation by humans [26, 27, 31].

We collected data mainly in BNP, with a few additional observations from adjacent managed forest. We used three permanent study plots in BNP (denoted as MS, N, W) totalling c. 130 ha [26, 33] and also other fragments of primeval oak-lime-hornbeam *Tilio-Carpinetum* or mixed *Pino-Quercetum* stands, which are the main habitats of Wood Warblers [34, 35]. The stands are a fine-grained mosaic of microhabitats within the broad habitat types that cover large areas of the forest [31, 36]. The multi-layered stands are composed of various tree species of diverse sizes, aged up to several hundred years old, dominated by hornbeam *Carpinus betulus*, lime *Tilia cordata*, oak *Quercus robur*, spruce *Picea abies*, maple *Acer platanoides* and pine *Pinus sylvestris*, which occur in varying proportions between oak-lime-hornbeam and mixed stands*.* Fallen and standing dead wood is moderately common or abundant [31, 37].

### Study species

We focused on a ground-nesting songbird, the Wood Warbler *Phylloscopus sibilatrix,* and mainly *Myrmica* ant species that also raise their broods on the forest floor. Wood Warblers are small (c. 10 g) migratory songbirds that winter in equatorial Africa and breed in temperate European forests [38]. The birds build dome-shaped nests of woven grass, tree leaves and moss, lined with animal hair. The nests are usually well-hidden among leaf litter and sparse vegetation on the ground [39].

A previous study in BNP found that c. 30% of Wood Warbler nests contained mostly *Myrmica ruginodis* and *M. rubra* broods, which were located within the nest walls [25]. Both *Myrmica* species are abundant ant species in many parts of Eurasia [40, 41]. Their colonies contain from tens to thousands of workers, and can be found on the forest floor. The densities of ant colonies can be limited by the availability of warm nest locations that depend on exposure to the sun in cool, temperate woodlands [21, 42, 43]. Therefore, access to active nests of birds, heated from within by the owners, could be particularly important for these insects, which require nest temperatures above 15 °C for the development of their broods [16, 17, 29, 30].

### Placement of bird nests

We searched for Wood Warbler nests on a daily basis from late April until mid-July in 2018–2020 by following birds, mainly during nest-building. The nesting period of Wood Warblers largely overlapped with the peak of brood-rearing by *Myrmica* (and *Lasius*) ants, preceding the ants’ nuptial flights in July–September [17, 44, 45]. We inspected each bird nest every 1–6 days to establish the dates of egg-laying commencement (when the 1st egg was laid), hatching (assigned as day 0 of the nestlings’ age), nestlings vacating the nest (fledging), or nest failure. The breeding attempts of Wood Warblers usually lasted for 32 days from the first egg being laid until all young fledged, and included 5–7 days of egg-laying, 13 days of egg incubation and 12–13 days of chick-rearing [34, 38]. Nest failure was primarily due to predation, which is the main cause of the Wood Warbler nest losses in BNP [34, 39, 46].

To check if Wood Warblers preferred to nest near specific habitat features that might potentially hold ant colonies, we took descriptions of 187 Wood Warbler nest locations and compared them to 187 controls. The controls were points chosen haphazardly c. 30 m from the bird nests. The direction of each control was appointed by an observer turning around and stopping on a command from a second observer, who had no eye-contact with the partner. The distance of 30 m was measured in strides, always by the same observer. On a few occasions, a control fell on a road or in a meadow, which were unrepresentative of Wood Warbler nest-locations. Therefore, the procedure was repeated in such cases, and a new control was selected.

The descriptions of the Wood Warbler nests and the controls included the presence of the nearest tussock of vegetation (fern, grass or sedge), fallen tree branch and/or larger tree log, which were the distinctive features on the forest floor that might also potentially hold ant colonies [40, 41, 47]. Each nest could have multiple features recorded. The minimum diameter of the recorded deadwood branches was 1 cm, which was the minimum branch size that contained an ant colony in this study area (M. Maziarz, pers. obs.). Other fallen deadwood with a minimum diameter of 5 cm was defined as ‘tree log’. We used the two categories of deadwood to consider their potential differences in ‘quality’ as nest locations for ants due to the potentially varying microclimates [48]. We measured the distance to all nearest features (tussock, branch and/or log) that were present within three metres of the rim of a bird nest or from the control, i.e. within a reachable distance for *Myrmica* (and *Lasius*) ants (M. Maziarz, pers. obs. [44, 49]).

### The density and placement of ant colonies

To test whether Wood Warblers selected forest patches with higher densities of ant colonies, and to establish the placement of ant colonies, in 2018–2020 we searched for ant colonies on the forest floor. We defined an ant colony as a group of ants, including workers and/or a queen that were accompanying larvae or pupae, occupying a ‘nest’ structure other than a bird nest [44].

We conducted searches for ant colonies within 133 pairs of 3 × 3 m sample squares, with one of each pair centred on a Wood Warbler nest and the other on the haphazardly allocated control point, located c. 30 m from the nest (see above). To avoid disturbing Wood Warblers and exposing them to nest predators, we searched for ant colonies only after the Wood Warbler chicks had fledged or the birds’ breeding attempts had failed naturally.

The surveys entailed careful inspection of the forest litter to find all ant colonies within the squares. We treated colonies as present if a brood (larvae/pupae) could be seen above the ground surface, enabling the precise location of each ant colony. First, we marked all colonies found within a plot, and then we measured the distances between them. We took descriptions of the location of all ant colonies found within the plots, and collected specimens of ant workers for later identification. Where any ant broods occurred less than 55 cm apart, we treated these as one ant colony to avoid potential multiple counts of the same colony. In those situations, we used the description of the brood location that was found first.

### Inspection of the Wood Warbler nests for ant broods

To establish the presence of ant broods within the walls of Wood Warbler nests, in 2018–2020 we collected 260 bird nests from the field after the chicks had fledged or the breeding attempt had failed, but only if the nest structure remained intact. We placed each nest into a sealed and labelled plastic bag, which contained information on the collection date and the nest identification number. To ascertain the presence or absence of ant broods in the bird nests, for those nests collected in 2018, we carefully pulled the nesting material apart and searched for ant larvae or pupae amongst it [25]. If an ant brood was present, we collected five to ten ant workers from each bird nest into labelled tubes filled with alcohol, for later species identification.

For bird nests collected in 2019–2020, we automated nest examination by first extracting invertebrates from them using a Berlese–Tullgren funnel. Each Wood Warbler nest was covered with fine metal mesh and placed c. 15 cm under the heat of a 40 W electric lamp. All specimens, including ants, were caught in 100 ml plastic bottles containing 30 ml of 80% ethanol, installed under each funnel. Specimen extraction with the Berlese–Tullgren funnel usually took three days per nest. Next, we checked the nesting material as described above to ensure that no specimens remained. All ant specimens were then separated from other invertebrates caught in tubes and identified to species level.

### Data analysis

#### Observed and expected frequency of ant broods within bird nests

To test for non-random presence of ant broods in the Wood Warbler nests, we compared the observed and expected proportions of bird nests containing ant broods. The observed proportions were calculated separately for each year in 2018–2020.

To obtain the expected proportions we performed simulations of the number of cases when an individual random point fell within an 8 cm radius of the centre of a 3 × 3 m square, representing an ant brood within a typical Wood Warbler nest, while a random point outside of the 8 cm radius represented an ant colony outside of a bird nest. The limit of 8 cm corresponded to the average radius of a Wood Warbler nest (M. Maziarz, pers. obs.). The simulations were based on a uniform distribution function that generated random deviates. We calculated an expected mean proportion of bird nests with ant broods and the 95% confidence intervals using bootstrapping (40,000 replications) in the ‘boot’ package in R [50, 51].

We repeated these simulations a further five times with an incrementally increasing number of random points allocated to the survey square in each replication, up to a maximum of six hypothetical ant colonies (the maximum number recorded within a real sample plot in BNP; Additional file 1: Table S1). To match the classification of a single ant colony in the field (see above), we set a minimum allowable distance of 55 cm between simulated ant colonies.

We multiplied the derived expected and observed proportions by 100 to obtain percentages.

#### Nest-site selection by the birds

According to our preliminary analyses, a tussock of vegetation, a fallen branch, or a tree log was present within three metres of 80–98% of the Wood Warbler nests and 79–98% of controls. Therefore, to provide a more sensitive test of the birds’ preference for nesting close to any of these habitat features, we considered them as present only if they were within 10 cm from the edge of a typical Wood Warbler nest of an 8 cm radius (M. Maziarz, pers. obs.), i.e. within 18 cm from the centre of a bird nest or a control.

To test if birds selected nest-sites close to a tussock of vegetation, a fallen branch and/or a tree log, we compared the frequency of any of these three features at 187 bird nests and 187 controls, using Chi^2^-tests with Yates' continuity correction. We did the comparisons separately for each of the three categories of habitat features, and included pooled samples from all years (2018–2020), as separate annual calculations were prevented by small sample sizes of bird nests or controls located at tree logs.

To check if Wood Warblers preferred to nest in areas more densely populated by ants, we compared the number of ant colonies recorded on the sample plots (3 × 3 m) centred on 133 Wood Warbler nests with the number of ant colonies found on 133 control plots. We tested the differences using a generalised linear model (GLM) with a Poisson error distribution and log-link function. The model contained the number of ant colonies as a response variable, and fixed covariates of year and the type of sample plot (bird nest vs control), with both covariates set as factors. An interaction term between year and plot type was insignificant in an initial model (AIC = 821.5), so it was removed from the final approach.

In all analyses, we treated the nest site choice of birds as independent each year because Wood Warblers show a low return rate to their breeding grounds in Continental Europe (up to 5% in Białowieża Forest; [52]), so it was unlikely that the nests found in different years could belong to the same birds.

#### Ant colony placement

To assess which features on the forest floor were used by ants for raising their broods, we calculated the annual proportions of ant colonies recorded in the most frequent categories of: fallen branch (≥ 1 and < 5 cm diameter), tree log (≥ 5 cm diameter), tussock of vegetation, and additionally deciduous tree-leaves, moss, bird nest, and ‘other’ uncommon locations, such as fallen spruce bark, standing tree or stump, soil, tree root, bracket fungus, spruce cone, or molehill. Colony frequency in each category was calculated separately for each year and for the two types of sample plots (centred on Wood Warbler nests and control locations). If an ant colony was located under two or more of the different features, the record was divided between the categories. For example, if an ant colony was found under a fallen branch lying on a tussock of vegetation, or under moss on a branch or log, each feature category received a score of 0.5. The sample sizes of ant colonies found in the sample plots at bird nests and control locations were respectively 73 and 53 in 2018, 64 and 58 in 2019, and 110 and 96 in 2020.

#### Prevalence of ant broods within bird nests in relation to bird nest placement

To test whether bird nests that were situated close to potential locations of ant colonies, such as tussock of vegetation, fallen branch or tree log, contained ant broods more often than the nests placed away from these features, we compared the frequencies of ant broods in these nests using Chi^2^- tests with Yates' continuity correction.

To check if the likelihood of an ant brood occurring in a Wood Warbler nest was higher in plots containing a greater number of ant colonies, we used a GLM with binomial error distribution and ‘logit’ link function. The model contained the presence or absence of an ant brood in a bird nest (respectively n = 41 and 88 bird nests) as a response variable, and a fixed covariate of the number of ant colonies on a sample plot. Preliminary analysis showed an insignificant effect of an interaction term between year (set as a factor) and the number of ant colonies (AIC = 167.1), or the fixed effect of year alone (AIC = 163.3), so both terms were dropped from the final model.

In the analyses, we assumed that the sample sizes included independent ant colonies each year because both the Wood Warbler nests and control locations always fell in different forest localities, which determined the searches for ant colonies each year. As such, it was unlikely that the same ant colonies would be tested repeatedly between years.

#### ‘Thermal’ factors influencing the occurrence of ant broods in bird nests

To test which of the multiple variables related to the thermal activity of the birds during their breeding cycle progression, or weather conditions, may influence the likelihood of ant colonies occurring in Wood Warbler nests, we performed model selection based on the AICc criterion [53].

The variables included in the models were: the nest stage (egg-laying, incubation or early nestling stage vs late nestling stage, when chicks were 5 days or older until fledging or failure), the mean daily ambient temperature (5-day average) and the daily sum of rainfall (5-day sum) preceding the Wood Warbler nest failure or chicks’ fledging, the delay of bird nest collection from the field (the number of days following fledging of the chicks or nest failure until the nest was collected), and year to account for the annual variation in all variables (Additional file 1: Table S2; for detailed description of the variables see Additional file 1: Table S3).

To assess weather conditions, we extracted mean daily ambient temperatures and daily sums of rainfall from the meteorological station in Białowieża village, situated approximately 1–6 km from the study areas. For each nest, we calculated the 5-day average temperature and the 5-day sum of rainfall preceding the date of the nest failure or chicks’ fledging. The date of nest failure or chicks’ fledging was a mid-date between the last visit of an observer when the nest was still active and the next visit, when nest failure or chick fledging had occurred (Additional file 1: Table S3). We used the 5-day periods to test the effect of weather conditions on the ants’ colonisation because we were unable to define the exact date of ants relocating their broods into active nests of birds (currently occupied by the nest owners). Confirming the presence of ant broods within the structure of bird nests was possible only after dissecting the nesting material [25], and so it was unethical until after birds had vacated the nests. We presumed that the 5-day period preceding nest failure or chicks’ fledging would be the most sensitive for determining the weather impact on ant colonisation: long enough for ant workers to respond to weather conditions and relocate their broods into bird nests [16, 54], or to stay within the bird nests if ant colonisation had already taken place.

For model selection, we created a global model, which was a GLM with a binomial error distribution and ‘logit’ link function. The model contained a response variable of the presence or absence of an ant brood in a bird nest (respectively n = 56 and 204 nests), and fixed covariates of nest stage and year set as factors, the delay of bird nest collection from the field, and also the main effects and the interaction term between the mean daily ambient temperature and the daily sum of rainfall. The remaining interactions between year and the nest stage, and between year or the nest stage and other continuous covariates, were insignificant in prior tests (AIC ≥ 262.4), so they were dropped from the model selection. We performed model selection based on the global model using the dredge function in the MuMIn package in R [55], where the null model contained only the intercept.

The coefficients and 95% confidence intervals (CI) of the variables were assessed from model averaging in the MuMIn package across the top candidate models with Δ AICc < 2.

We performed all statistical analyses in R version 4. 0. 2 [56].

## Results

### Is presence of ant broods within bird nests a non-random phenomenon?

The percentages of Wood Warbler nests containing ant broods amounted to 24% of 92 nests in 2018, 10% of 68 in 2019, and 27% of 100 in 2020. The broods and associated workers found within bird nests were mostly *M. ruginodis* (71% of 55 identified samples across all years), *M. rubra* (18%) and rarely *Lasius platythorax* (11%).

The observed prevalence of ant broods in the warbler nests was non-random; the percentages of nests containing ant broods were by one or two orders of magnitude greater than the expected random values derived from the simulations, depending on the number of hypothetical ant colonies in the plots (0.2–1.2%; Additional file 1: Table S4).

### Do Wood Warblers select nest sites that enhance colonisation by ants?

**Fig. 1:**
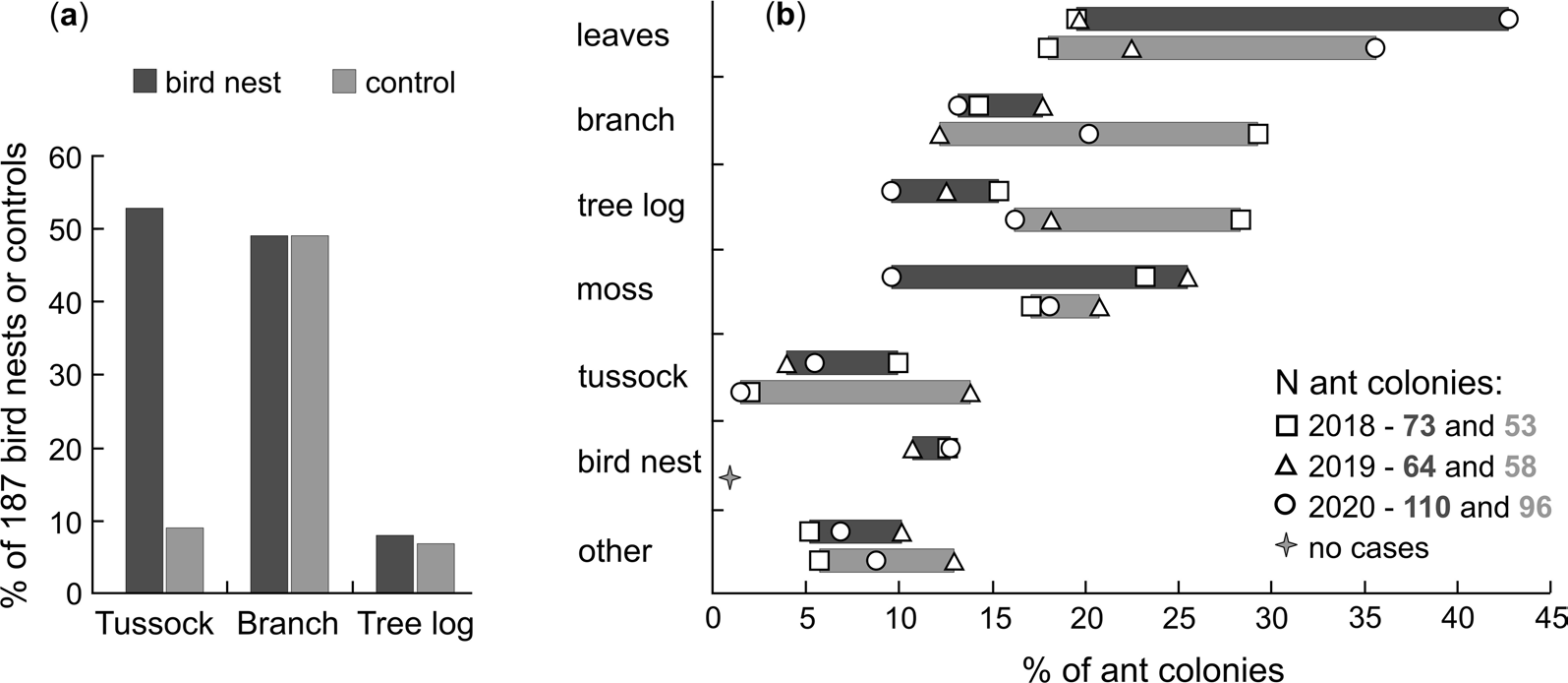
**a** The percentage of Wood Warbler *Phylloscopus sibilatrix* nests and control points at which a tussock of vegetation (grass, sedge or fern), fallen branch (≥ 1 and < 5 cm diameter), or a tree log (≥ 5 cm diameter) was present, and **b** the percentage of ant colonies using different features near to bird nests and controls in 2018–2020. ‘Other’ features used by ants included: decayed wood, fallen spruce bark, a standing tree or stump, soil, tree root, bracket fungus, spruce cone, molehill

Wood Warblers showed a preference for nesting in the vicinity of a tussock of vegetation, which was more frequent than at control locations (Chi^2^ with Yates' continuity correction = 82.0, df = 1, *P* < 0.001; Fig. 1a). However, there was no preference for nesting next to a fallen branch or tree log (Chi^2^ with Yates' continuity correction < 0.4, df = 1, *P* > 0.8).

Also, the birds nested in localities with only slightly higher densities of ant colonies than in control locations, and this was consistent between years, despite significant annual variation in ant colony densities on the forest floor (Additional file 1: Tables S1, Table 1).

**Table 1:** Results of a Generalised Linear Model with Poisson error distribution and log-link function testing the difference in the number of ant colonies (response variable) recorded on sample plots distributed around Wood Warbler *Phylloscopus sibilatrix* nests and control locations (n = 133 each) in 2018–2020

Variable	Estimate	SE	CI 2.5%	CI 97.5%
**Intercept**	** 0.339**	**0.099**	** 0.14**	**0.53**
Sample plot (control)	− 0.166	0.094	− 0.35	0.02
Year (2019)	0.131	0.125	− 0.12	0.38
**Year (2020)**	** 0.662**	**0.113**	** 0.44**	**0.89**

The model included main effects of the type of the sample plot (bird nest or control) and year as fixed covariates. Significant relationships, where 95% confidence intervals (CI) did not overlap with 0, are marked with bold font

Residual deviance = 250.4, df = 262, AIC = 818.9

The nest site selection of Wood Warblers did not overlap with the most frequent usage of features by ant colonies on the forest floor. The ants situated their broods mainly within or under deciduous tree-leaves, tree branches or logs, and/or mosses, while tussocks of vegetation were used rarely (Fig. 1b). The majority of ant colonies found in survey squares were *M. ruginodis*, which was identified in 67% of 245 colonies surrounding the Wood Warbler nests and in 62% of 207 colonies around control locations. Another common species was *M. rubra*, found in 30% of ant colonies around bird nests and in 32% around control locations. Occasional colonies belonged to *L. platythorax* (respectively 3% and 4%)*, L. brunneus* (c. 1% each) and *Temnothorax crassispinus* (one bird nest plot)*.*

The placement of Wood Warbler nests had no effect on the occurrence of ant broods within bird nests. Ant larvae or pupae were equally likely to occur in warbler nests situated under a tussock or placed further from it (respectively 21% and 29%; Chi^2^ with Yates' continuity correction = 0.9, df = 1, *P* = 0.33; Additional file 1: Table S5). Similarly, there was no relationship between ant colonisation of nests with or without a tree branch (respectively 21% and 28%) or a tree log (respectively 31% and 25%; Chi^2^ with Yates' continuity correction < 0.8, df = 1, *P* > 0.3; Additional file 1: Table S5). The likelihood of an ant brood occurring in a Wood Warbler nest was also unrelated to the number of ant colonies in the plots surrounding bird nests (Table 2), which averaged to 2.1 (SD = 1.1, n = 41) for the bird nests containing ant broods and 1.8 (SD = 1.2, n = 88) for the nests without them.

**Table 2:** Results of a Generalised Linear Model with binomial error distribution and ‘logit’ link function testing the likelihood of an ant brood occurring within a bird nest (response variable: ant brood present or absent) in relation to the number of ant colonies recorded on sample plots around bird nests (covariate)

Variable	Estimate	SE	CI 2.5%	CI 97.5%
**Intercept**	**− 1.293**	**0.383**	**− 2.08**	**− 0.57**
Number of ant colonies	0.273	0.166	**− **0.05	0.61

A significant relationship, where 95% confidence intervals (CI) did not overlap with 0, is marked with bold font

Residual deviance = 158.6, df = 127, AIC = 162.6

### Do ‘thermal’ conditions promote ant colonisation of bird nests?

**Fig. 2:**
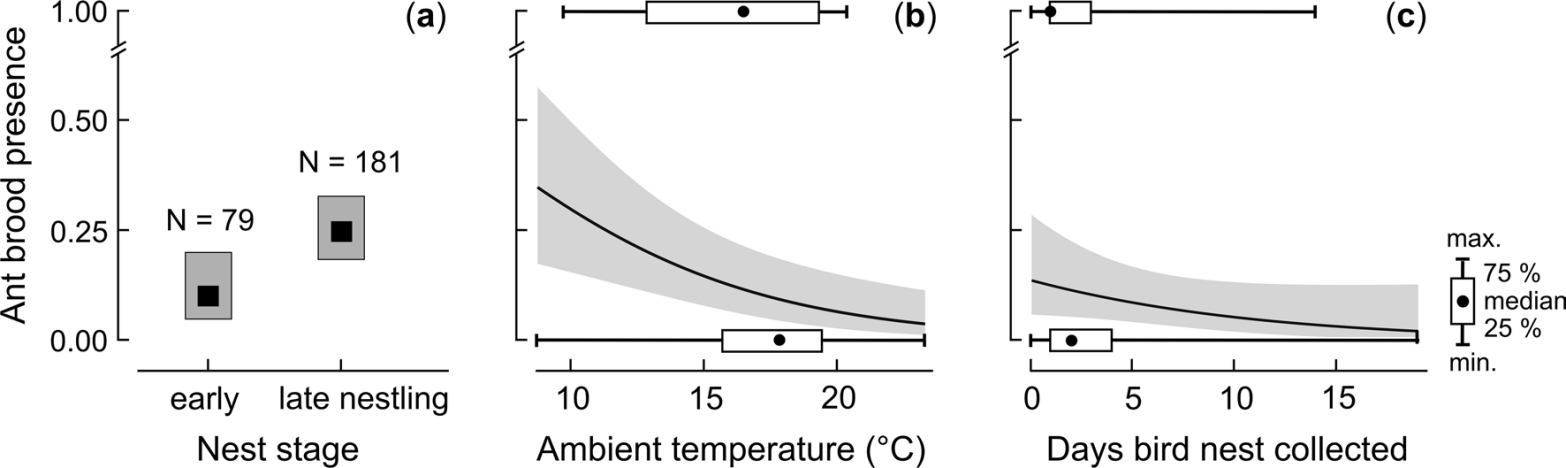
The likelihood of an ant brood occurring in a Wood Warbler nest: brood absent (0; n = 204) or brood present (1; n = 56), in relation to **a** the stage of bird nest, **b** the mean daily ambient temperature (5-day average) preceding nest failure or fledging of chicks, and **c** the number of days following nest failure or chicks’ fledging since the bird nest was collected. The mean probabilities (**a** black squares or **b**, **c** lines) and 95% confidence intervals (grey ranges) were assessed from the top model with Δ AICc = 0.00 (Table 3). Horizontal box-plots show median, 25–75%, and min–max **b** ambient temperature or **c** the delay in bird nest collection from the field for the nests without (0) and with (1) ant broods

Ant broods were more likely to occur in bird nests collected after the late nestling stage, which had contained large chicks prior to fledging or predation, rather than the early nest stage, when nests had failed during egg laying, incubation or the early nestling period (Fig. 2a, Additional file 1: Table S6). The likelihood of ant broods occurring in a bird nest also increased significantly with the decreasing ambient temperature in the days preceding the nest failure or fledging of chicks (Fig. 2b, Additional file 1: Table S7). Both the nest stage and ambient temperature were retained in all top candidate models assessing the likelihood of an ant brood occurring within a bird nest (Δ AICc < 2), and were significant in model-averaging (the 95% confidence intervals of these variables did not overlap with 0; Tables 3 and 4).

**Table 3:** The results of model selection using the corrected Akaike Information Criterion (AICc) testing the likelihood of an ant brood occurring (present or absent) in a Wood Warbler nest (response variable) in relation to covariates of the stage of a bird nest, ambient temperature and rainfall preceding fledging of chicks or nest failure, the delay of bird nest collection from the field, and year

Models	df	log-lik	AICc	ΔAICc	Weights
Nest stage^a^ + temperature^b^ + delay of bird nest collection^c^	4	− 125.5	259.2	0.00	0.205
Nest stage + temperature + year^d^	5	− 125.0	260.3	1.16	0.115
Nest stage + temperature	3	− 127.3	260.6	1.44	0.100
Nest stage + temperature + rainfall^e^ + temperature × rainfall + delay of bird nest collection	6	− 124.3	261.0	1.83	0.082
Nest stage + temperature + rainfall + delay of bird nest collection	5	− 125.4	261.0	1.85	0.082

Shown are the top Generalised Linear Models with a binomial error distribution and ‘logit’ link function, where Δ AICc was < 2. The null model contained an intercept only

^a^Early nest stage versus late nestling stage; ^b^5-day average of daily means; ^c^number of days following nest failure or chicks’ fledging; ^d^2018–2020; ^e^5-day sum of daily sums

**Table 4:** The results of model-averaging across the top candidate models with Δ AICc < 2 (Table 3) investigating the likelihood of an ant brood occurring in a Wood Warbler nest in relation to the stage of bird nest, ambient temperature and rainfall preceding nest failure or fledging of chicks, the delay of bird nest collection from the field, and year

Variable	Estimate	SE	CI 2.5%	CI 97.5%
Intercept	0.935	1.237	− 1.499	3.369
Delay of bird nest collection	− 0.105	0.062	− 0.227	0.017
**Nest stage (large nestlings)**	** 1.149**	**0.469**	** 0.227**	** 2.072**
**Ambient temperature**	** − 0.168**	**0.074**	** − 0.313**	** − 0.023**
Year (2019)	− 0.939	0.478	− 1.881	0.003
Year (2020)	− 0.184	0.376	− 0.924	0.557
Rainfall	0.062	0.099	− 0.132	0.256
**Ambient temperature × rainfall**	** − 0.008**	**0.005**	** − 0.018**	** − 0.003**

Shown is conditional average. Significant relationships, where 95% confidence intervals (CI) did not overlap with 0, are marked with bold font

**Fig. 3:**
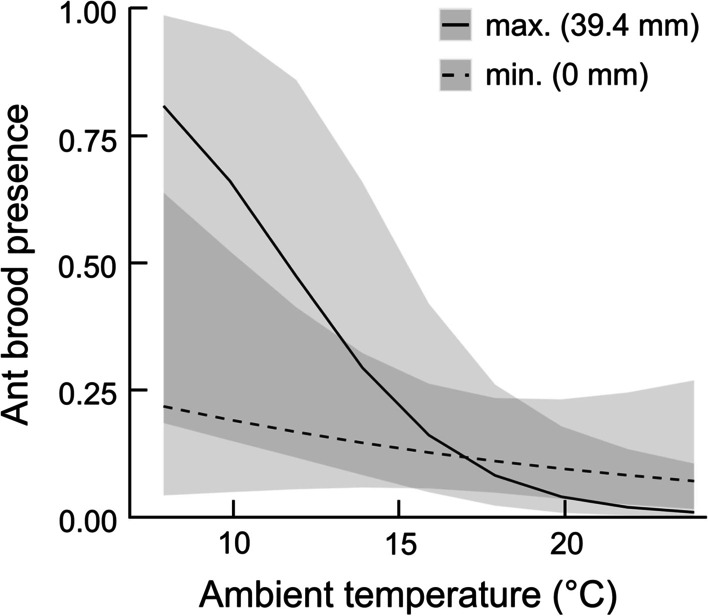
The likelihood of an ant brood occurring in a Wood Warbler nest: brood absent (0; n = 204) or brood present (1; n = 56), in relation to the mean daily ambient temperature (5-day average) and the sum of rainfall (5-day sum) preceding nest failure or fledging of chicks. The mean probabilities for the minimum and maximum sum of rainfall and the 95% confidence intervals (grey shades) were assessed from the model with Δ AICc = 1.83 (Table 3)

More rainfall during cold weather in the days preceding nest failure or chick fledging also increased the chances of an ant brood occurring within a bird nest (Fig. 3, Additional file 1: Table S7). A negative interaction between the sum of rainfall and the mean daily ambient temperature was included in the model with Δ AICc < 2, and this was also significant in the averaged model (Tables 3 and 4).

In contrast, the delay of bird nest collection from the field had little negative effect on the occurrence of an ant brood within a bird nest (Fig. 2c, Additional file 1: Table S7); despite this variable being included in the three models with Δ AICc < 2, it became insignificant in model averaging (Tables 3 and 4). The proportion of Wood Warbler nests containing ant broods was lowest in 2019 (see above), but the effect of year on the likelihood of an ant brood occurring in a bird nest became insignificant in the averaged model (Tables 3 and 4).

## Discussion

### Attraction between nesting birds and ants

This study is the first to show that the colonisation of bird nests by ants raising their own broods is a non-random phenomenon, which occurs much more frequently than expected by chance, indicating interspecific attraction. Contrary to our expectations, the colonisation appeared to be driven solely by the ants’ attraction to the bird nests, as there was no evidence for the attraction of Wood Warblers to ant colonies through the birds’ nest-site choice. The birds’ selection to nest near tussocks overlapped little with the ants’ placement of their colonies on the forest floor. Additionally, birds did not seem to select forest localities with higher densities of ant colonies, and the presence of ant broods within bird nests did not depend on the nest placement by the Wood Warblers. Thus, the non-random occurrence of ant broods within bird nests was unlikely to be driven by the nest-site selection of Wood Warblers.

The habitat features we recorded (tussock of vegetation, fallen tree branch and/or tree log) were distinctive habitat elements that could be used by birds for locating ant colonies on the forest floor. In contrast, deciduous tree-leaves (which were used by ants most often for rearing their broods) covered most of the area in the deciduous or mixed forest stands. As such, we assumed that the presence of fallen tree leaves would provide no specific cues for birds to select nest sites nearby ant colonies.

The haphazard selection of control locations should reflect the approximate frequency of tussocks, fallen tree branches or tree logs on the forest floor, as similar values were also obtained for 41 blindly-selected GPS locations (of 18 cm radius) examined in 2018, where a tussock was present at 5%, a branch at 56%, and a log at 10% (Maziarz et al., unpubl. data). These results and unpublished information indicated that the much more frequent occurrence of tussocks at the Wood Warbler nests compared to the controls 30 m from bird nests (Fig. 1a), or blindly selected GPS locations, was most likely reflecting a true preference of birds to nest near tussocks. The nest site selection of Wood Warblers near tussocks was probably beneficial in concealing their nests from predators, which constitute the major threat to these birds [46, 57, 58].

The slightly higher number of ant colonies nearby bird nests than controls probably resulted from attraction of ants to the former, rather than birds selecting areas with a higher abundance of ant colonies. Due to methodological constrains (see “Methods” section), we were unable to establish the number of ant colonies at bird nests during the nest building stage. By the time the nests failed or young had fledged, some ant colonies could move closer to bird nests, and the colonisation of bird nests by ants indicated that this indeed happened.

The pressure to choose specific nest sites that enhance colonisation by ants should be low for the birds nesting in the areas with high densities of ant colonies on the forest floor, as any random placement of bird nests would likely provide ants with relatively easy access to bird nests. Despite the lack of apparent attraction of birds to ant colonies, at least 86% of Wood Warbler nests, and also at least 65% of the control locations, were situated within c. 2 m of an ant colony (centre of a 3 × 3 m plot; Additional file 1: Table S1), so within an accessible distance for ants to relocate their broods ([44, 49], M. Maziarz, pers. comm.). Such a proximity of ant colonies to bird nests and control locations was possible due to the high density of ant colonies on the forest floor. Thus, nesting in close proximity of ant colonies probably facilitated the colonisation of bird nests by ants and released any selective pressure on birds to nest in specific sites that would enhance colonisation by ants.

Our assessment of the density of ant colonies, averaging 0.12–0.30/m^2^, was probably slightly underestimated as it included only the colonies where ant larvae or pupae were visible on the ground surface, and also omitted potential colonies that were less than 55 cm from the focal ant colony (see “Methods” section). Despite this, the densities of ant colonies that we recorded in the undisturbed forest of BNP remained comparable to the overall densities of *Myrmica* and *Lasius* nests in mature (c. 90–150 years old) pine stands in the managed part of the Białowieża Forest, and also elsewhere in Poland (0.13–0.34/m^2^; [59]). Although the ant densities found in these old growth stands in Poland were higher than in spruce stands in Russia, near Moscow [47], they were several times lower than in some other habitats in Hungary and northwestern USA, where ant species composition also differed [60, 61]. This suggests that relatively high densities of ant colonies are probably common across a range of habitats, providing ants with easy access to ground-nesting birds and promoting cohabitation between the two groups.

Despite the lack of clear attraction of Wood Warblers to ant colonies in our results, birds might still benefit from a close association with the predatory ants, e.g. through reduced infestation with nest-dwelling ectoparasites or disease vectors [10–13, 62]. Further investigations would be desirable to clarify the potential advantages for the birds from the presence of ants within or nearby their nests.

### The impact of ‘thermal’ conditions on ant broods’ presence within bird nests

As expected, the likelihood of an ant brood occurring within a Wood Warbler nest increased significantly in the late nestling period. Cool weather further enhanced colonisation of Wood Warbler nests by ants, and also when higher rainfall coincided with low ambient temperatures. These findings supported the hypothesis that ants colonised bird nests to raise their own broods in a more advantageous microclimate than in the ants’ own nests elsewhere on the forest floor [20]. It is possible that longer exposure of nests containing older nestlings increased the potential colonisation of bird nests in the later nestling stage, relative to earlier nest stages. Despite this, the largest temperature disparities between bird nests and the ants’ own nests at the later nestling stage [20], could drive the decision of ant workers to relocate their broods into the much warmer bird nests, containing older nestlings. The experiments with heated and unheated artificial nests that mimicked the natural active and inactive nests of birds showed that the presence of heat within attracted ants for rearing own broods [20]. Temperatures of 20–25 °C, which are preferred by *Myrmica* and *Lasius* ants for optimal growth and development of their larvae or pupae [16, 29, 30], could occur only in occupied Wood Warbler nests that were warmed up by older nestlings [20]. Meanwhile, similar conditions were unachievable in the ants’ own nests under the same ambient temperatures of 17–21 °C. Higher rainfall during cool weather probably further increased the microclimate disparity between the birds’ nests and the ants’ own nests, perhaps by increasing the humidity in bird nests and/or by flooding the ants’ original nest locations, forcing the workers to relocate their broods [21].

As the attractiveness of bird nests for the ants raising their own broods seemed to depend on ambient temperature and rainfall, weather conditions could act as agents of the interactions between the two groups of animals. Progressive climate warming that affects many areas of the Northern Hemisphere, including BNP [63, 64], might therefore pose a previously unrecognised threat to these poorly known interactions between birds and invertebrates. In warmer springs, the disparity between the microclimate of bird nests and other nest locations of ants would become smaller, and so the benefits for the insects to raise their broods within bird nests would be reduced. Increasing ambient temperatures would therefore relax the selective pressure on ants to colonise warm nests of birds, leading to the disappearance of the interspecific relationships.

Contrary to expectations, an increasing delay in collecting bird nests from the field only slightly reduced the likelihood of finding an ant brood within them, in both early and late nesting stages (the interaction between the nest stage and the delay of nest collection from the field was insignificant in the initial model, see “Methods” section). This was surprising, as bird nests cool soon after being vacated, and the nest temperatures quickly level to ambient temperatures [20]. Despite the loss of the advantage of using warmer nest locations than elsewhere, some ant workers apparently decided to keep their broods within bird nests for up to almost two weeks (Fig. 2c). Such a decision by ants, to remain within the Wood Warbler nests, might reflect the higher energetic costs of relocating the broods to new locations, which could outweigh the potential benefits. A previous study showed that, compared to outside, the mean daily temperatures of vacant (empty) Wood Warbler nests were similar to the ants’ own nests elsewhere [20]. As such, the thermal advantages of using other nest locations on the forest floor were probably comparable to that of vacant nests of birds. However, it is also possible that an easy access to food, e.g. in a form of other nest-dwelling invertebrates, bird faeces and other debris [21, 65] may encourage ants to stay within the bird nest.

### The overlooked but potentially important ecological links

Due to limited research on the associations between nesting birds (or mammals) and nest-dwelling invertebrates, the information on their prevalence is often fragmentary. For example, the sparse data for the proportion of ant broods within bird nests suggests large inconsistencies between regions, also limiting further conclusions on how widespread this phenomenon may be [12, 25]. The current study, carried out in the primeval stands of BNP, revealed that ant broods were present within 10–27% of the Wood Warbler nests. These values were comparable to the 20–30% recorded previously in BNP in 2004–2017 [25]. The proportions found in other studies involving the same or different bird and ant species in BNP or elsewhere were usually lower than in the current study, comprising 2–18% of inspected nests [24, 25, 62, 66]. Only Gibson et al. [12] found similar or much higher percentages of bird nests containing ant colonies, with up to c. 90% in the North American Midwest.

The reasons for these large inconsistencies in rates of ants within bird nests between bird species and regions are unknown. They might be biased by a varying intensity of data collection or methodologies, climatic conditions, habitat type, and/or the degree of shading that affect the availability of other potential nest sites for the ants [12, 24, 25, 42, 43]. Transformation and fragmentation of forests can affect the abundance and species composition of both ant and bird communities [59, 67, 68], and this might also potentially change the dynamic of the interspecific interactions between these two groups. Thus, information from both human-transformed and undisturbed habitats would be valuable to explain the differences between areas facing different anthropogenic pressures.

These poorly known associations between nesting birds and ants may be ecologically important. Access to warm nests of birds may provide ants with an advantage in temperate mature forests, like primeval stands of the Białowieża Forest, where the tree canopy is dense and ant nest locations, warmed by solar radiation, are limited [21, 42, 43]. In contrast to variable solar heating, bird nests are consistently warmed by breeding Wood Warblers for around four weeks, similar to the nesting period of many other songbirds across a range of habitats (e.g. [38]). As the breeding period of many birds largely overlaps with that of brood-rearing by *Myrmica* and *Lasius* ants (April–July; [17, 44, 45]), the insects can take advantage of warm bird nests during this critical period.

Although only some ant colonies would likely have access to bird nests in a given area, the facultative usage of these warm locations by ants may be crucial for promoting fitness of the individual colonies. Exploiting warm nests of birds may accelerate the development of the ants’ larvae or pupae, especially in cooler regions, where colonies are more temperature dependent than in warmer climates [29, 30]. Thus, birds building and warming their nests from within can act as thermal ecosystem engineers by providing important resources of nest sites for ants, or other invertebrates, that are reliant on ambient temperatures for raising their larvae or pupae [18–20].

Despite the fact that aggression of ants to nesting birds has frequently been reported (e.g. [62, 69, 70]), we observed no attacks by *Myrmica* or *Lasius* ants on the ground-nesting Wood Warblers in BNP, further supporting the existence of positive interactions between these two groups. However, more research would be needed to explain how the birds may prevent the nest-dwelling ants entering their nest cups (containing eggs or nestlings), and which factors might shift the behaviour of ants towards nesting birds from aggressive to non-aggressive. As the temperature of active nests of birds changes gradually from the nest cup to the nest rim [71], ants could place their larvae or pupae slightly further from the nest cup, where the optimal temperatures for incubating ant broods could be found. If true, ants and birds could avoid direct contact and any potential negative interactions.

The very widespread distribution and abundance of nest-building birds, and also mammals, and their associated temperature-dependent invertebrates suggests that similar associations may be much more common across cool regions of the World. These probable positive interactions between nesting vertebrates and various taxa of nest-dwelling invertebrates, including ants, may form intricate ecological networks, like those already studied among well-known pollinator, frugivore or ant-plant networks [3]. Therefore, further investigations would be desirable to explore the extent of the little known, but potentially important links between warm-blooded vertebrates and nest-dwelling invertebrates within ecological networks.

## Conclusions

The study demonstrates a non-random occurrence of ant broods within bird nests, which seems to be driven exclusively by the ants’ attraction to the nests. High densities of ant colonies on the forest floor may reduce the selective pressure on birds to nest nearby ant colonies and facilitate frequent colonisation of their nests by ants. The higher natural occurrence of ant broods within bird nests during the late nestling stage, when bird nests are warmest, and also in cool and wet weather, supports the hypothesis of ants seeking the greatest thermal benefits for raising their own broods. The study provides rare evidence to explain the poorly known interspecific interactions between warm-blooded vertebrates and nest-dwelling invertebrates. We highlight the need for more research to assess the extent of similar relationships that may be common across various taxonomic groups in relatively cool regions of the world.

## Supplementary Information

**Additional file 1: Table S1**. The number of ant colonies on sample plots (3 × 3 m) that were centred on Wood Warbler *Phylloscopus sibilatrix* nests and controls in 2018-2020. Shown are the total number of sample plots on which ant colonies were searched for, and the percentage of sample plots on which a minimum of one ant colony was found. An ant colony was defined as a group of ants (workers and/or a queen with larvae or pupae) occupying a nest structure other than bird nest. **Table S2**. The mean daily ambient temperature (5-day average) and daily sum of rainfall (5-day sum) preceding Wood Warbler nest failure or chicks’ fledging, and the delay of bird nest collection from the field in 2018-2020.** Table S3**. Variables used in selection of candidate Generalised Linear Models with a binomial error distribution and ‘logit’ link function, and the subsequent model averaging, testing the effect of the thermal activity of birds within their nests (nest stage, delay of bird nest collection from the field), weather conditions (temperature, rainfall) and year (2018-2020) on the likelihood of an ant brood occurring in a Wood Warbler nest.** Table S4**. The results of simulations and bootstrapping (40000 replications) showing the expected mean percentage and 95 % confidence intervals (CI) of a simulated ant brood (larvae or pupae) falling within a hypothetical bird nest (i.e. 8 cm from the nest centre), in relation to the number of ant colonies present in a sample plot (3 × 3 m) that was centred on a hypothetical bird nest. The distance of 8 cm from the nest centre corresponded to the approximate radius of a Wood Warbler nest. **Table S5**. The number of Wood Warbler nests with and without ant broods in relation to the proximity (within 18 cm from the nest centre) of a tussock of vegetation (grass, sedge or fern), fallen branch (≥ 1 and < 5 cm diameter), or a tree log (≥ 5 cm diameter), or where these features were absent near the bird nests.** Table S6**. The percentage and total number (n) of Wood Warbler nests containing ant broods in early nest stage (egg-laying or incubation, or < 5 days post-hatching) and late nestling stage (≥ 5 days post-hatching), in 2018-2020. **Table S7**. The mean daily ambient temperature (5-day average) and daily sum of rainfall (5-day sum) preceding bird nest failure or chicks’ fledging, and the delay of bird nest collection from the field for the Wood Warbler nests where ant broods were present or absent. The comparison includes early nest stage (egg-laying or incubation, or < 5 days post-hatching) and late nestling stage (chicks ≥ 5 days old).

## Data Availability

The datasets generated and/or analysed during the current study are available in the figshare repository, https://doi.org/10.6084/m9.figshare.16531164.v1 [72].
