# Supplementary material for: Interspecific attraction between ground-nesting songbirds and ants: the role of nest-site selection
Source: Front Zool. 2021 Sep 10;18:43. doi: 10.1186/s12983-021-00429-6 (PMC8434696; doi:10.1186/s12983-021-00429-6)
Supplement: Supplementary file 1 — Additional file 1: Table S1. The number of ant colonies on sample plots (3 × 3 m) that were centred on Wood Warbler Phylloscopus sibilatrix nests and controls in 2018-2020. Shown are the total number of sample plots on which ant colonies were searched for, and the percentage of sample plots on which a minimum of one ant colony was found. An ant colony was defined as a group of ants (workers and/or a queen with larvae or pupae) occupying a nest structure other than bird nest. Table S2. The mean daily ambient temperature (5-day average) and daily sum of rainfall (5-day sum) preceding Wood Warbler nest failure or chicks’ fledging, and the delay of bird nest collection from the field in 2018-2020. Table S3. Variables used in selection of candidate Generalised Linear Models with a binomial error distribution and ‘logit’ link function, and the subsequent model averaging, testing the effect of the thermal activity of birds within their nests (nest stage, delay of bird nest collection from the field), weather conditions (temperature, rainfall) and year (2018-2020) on the likelihood of an ant brood occurring in a Wood Warbler nest. Table S4. The results of simulations and bootstrapping (40000 replications) showing the expected mean percentage and 95 % confidence intervals (CI) of a simulated ant brood (larvae or pupae) falling within a hypothetical bird nest (i.e. 8 cm from the nest centre), in relation to the number of ant colonies present in a sample plot (3 × 3 m) that was centred on a hypothetical bird nest. The distance of 8 cm from the nest centre corresponded to the approximate radius of a Wood Warbler nest. Table S5. The number of Wood Warbler nests with and without ant broods in relation to the proximity (within 18 cm from the nest centre) of a tussock of vegetation (grass, sedge or fern), fallen branch (≥ 1 and < 5 cm diameter), or a tree log (≥ 5 cm diameter), or where these features were absent near the bird nests. Table S6. The percentage and total number (n) of [file 12983_2021_429_MOESM1_ESM.pdf]

Additional file 1

Title: **Interspecific attraction between ground-nesting songbirds and ants: the role of nest-site selection**

Marta Maziarz [1], Richard K. Broughton [2], Luca Pietro Casacci [3], Grzegorz Hebda [4], István Maák [5], Gema Trigos-Peral [1], Magdalena Witek [1]

[1] *Museum and Institute of Zoology, Polish Academy of Sciences, Wilcza 64, 00-679 Warsaw, Poland*

[2] *UK Centre for Ecology & Hydrology, Maclean Building, Benson Lane, Crowmarsh Gifford, Wallingford, OX10 8BB, UK*

[3] *Department of Life Sciences and Systems Biology, University of Turin, Via Accademia Albertina 13, 10123 Turin, Italy*

[4] *Institute of Biology, University of Opole, Oleska 22, 45-052 Opole, Poland*

[5] *Department of Ecology, University of Szeged, Közép fasor 52, 6726 Szeged, Hungary*

Table S1. The number of ant colonies on sample plots (3 × 3 m) that were centred on Wood Warbler *Phylloscopus sibilatrix* nests and controls in 2018-2020. Shown are the total number of sample plots on which ant colonies were searched for, and the percentage of sample plots on which a minimum of one ant colony was found. An ant colony was defined as a group of ants (workers and/or a queen with larvae or pupae) occupying a nest structure other than bird nest.

Year	Plot type	N plots		Number of ant colonies			
		total	% ant colony present	mean	SD	min	max
2018	Bird nest	49	87.8	1.5	0.9	0	4
	Control	49	65.3	1.1	1.1	0	5
2019	Bird nest	43	86.0	1.5	1.1	0	4
	Control	43	72.1	1.4	1.4	0	5
2020	Bird nest	41	97.6	2.7	1.1	0	5
	Control	41	92.7	2.3	1.4	0	6

Table S2. The mean daily ambient temperature (5-day average) and daily sum of rainfall (5-day sum) preceding Wood Warbler nest failure or chicks' fledging, and the delay of bird nest collection from the field in 2018-2020.

Year	N nests	Temperature (°C)			Sum of rainfall (mm)			Delay of nest collection (days)		
		mean	SD	min-max	mean	SD	min-max	mean	SD	min-max
2018	92	18.0	1.9	13.6-20.5	1.5	1.8	0.0-10.9	2.4	2.4	0-14
2019	68	17.8	2.7	8.7-23.3	4.9	7.2	0.0-30.9	6.1	4.8	0-19
2020	100	16.0	3.7	9.4-21.2	17.8	12.0	0.6-39.4	2.0	2.3	0-13

Table S3. Variables used in selection of candidate Generalised Linear Models with a binomial error distribution and ‘logit’ link function, and the subsequent model averaging, testing the effect of the thermal activity of birds within their nests (nest stage, delay of bird nest collection from the field), weather conditions (temperature, rainfall) and year (2018-2020) on the likelihood of an ant brood occurring in a Wood Warbler nest.

Variable	Description
Nest stage	Two categories: early nest stage (comprising the egg-laying, egg-incubation or post-hatching period, when chicks were < 5 days old), and the late nestling stage (when chicks were ≥ 5 days, until fledging or failure); during the egg-laying the nest was not warmed by birds (15 cases), and during the egg-incubation and post-hatching period, a single adult parent warmed up the nest walls from within (64 cases in total). While, in the late nestling stage, typically six or seven chicks warmed up the nest (181 cases). Consequently, the temperature of nest walls was highest in the late nestling stage (Maziarz et al. 2020).
Delay of bird nest collection	The number of days following fledging of the chicks or nest failure, when the nest was collected from the field. The date of fledging or nest failure was the mid-date between the last visit when the nest was still active and the next visit when nest failure or chick fledging were found, assessed with a precision of about three days for nest failures and one day for fledged nests.
Ambient temperature	Averaged mean daily ambient temperatures for the 5-day period preceding fledging of chicks or nest failure, when the weather conditions should be influential for detecting an ant brood in a Wood Warbler nest.
Rainfall	Sum of the daily rainfall sums for the 5-day period preceding the fledging of chicks or nest failure.
Year	2018, 2019 or 2020.

Table S4. The results of simulations and bootstrapping (40000 replications) showing the expected mean percentage and 95 % confidence intervals (CI) of a simulated ant brood (larvae or pupae) falling within a hypothetical bird nest (i.e. 8 cm from the nest centre), in relation to the number of ant colonies present in a sample plot (3 × 3 m) that was centred on a hypothetical bird nest. The distance of 8 cm from the nest centre corresponded to the approximate radius of a Wood Warbler nest.

The number of ant colonies	Mean	CI 2.5 %	CI 97.5 %
1	0.2	0.2	0.3
2	0.5	0.4	0.5
3	0.7	0.6	0.8
4	0.8	0.7	0.9
5	1.1	1.0	1.2
6	1.2	1.1	1.3

Table S5. The number of Wood Warbler nests with and without ant broods in relation to the proximity (within 18 cm from the nest centre) of a tussock of vegetation (grass, sedge or fern), fallen branch (≥ 1 and < 5 cm diameter), or a tree log (≥ 5 cm diameter), or where these features were absent near the bird nests.

Feature		Ant brood in bird nest		Total
		present	absent	
Tussock	present	19	70	89
	absent	24	59	83
Branch	present	18	66	84
	absent	25	63	88
Tree log	present	4	9	13
	absent	39	120	159

Table S6. The percentage and total number (n) of Wood Warbler nests containing ant broods in early nest stage (egg-laying or incubation, or < 5 days post-hatching) and late nestling stage (≥ 5 days post-hatching), in 2018-2020.

Year	Early nest stage		Late nestling stage	
	% with ant broods	n	% with ant broods	n
2018	9.1	22	28.6	70
2019	10.7	28	10.0	40
2020	17.2	29	31.0	71
Total	12.7	79	25.4	181

Table S7. The mean daily ambient temperature (5-day average) and daily sum of rainfall (5-day sum) preceding bird nest failure or chicks' fledging, and the delay of bird nest collection from the field for the Wood Warbler nests where ant broods were present or absent. The comparison includes early nest stage (egg-laying or incubation, or < 5 days post-hatching) and late nestling stage (chicks ≥ 5 days old).

Nest stage	Ant brood	N nests	Temperature (°C)			Sum of rainfall (mm)			Delay of nest collection (days)		
			mean	SD	min-max	mean	SD	min-max	mean	SD	min-max
Early	present	10	14.2	2.8	9.7-19.9	10.1	9.9	0.9-25.3	4.2	2.8	0-11
	absent	69	15.7	3.2	8.7-21.5	9.6	11.8	0.0-39.4	5.7	4.5	0-19
Late	present	46	16.9	2.9	12.1-20.3	8.8	10.2	0.0-39.0	1.8	2.3	0-14
	absent	135	18.3	2.4	12.2-23.3	8.1	11.2	0.0-39.4	2.4	2.8	0-16
